# Consent requirements for research with human tissue: Swiss ethics committee members disagree

**DOI:** 10.1186/s12910-018-0331-0

**Published:** 2018-11-26

**Authors:** Flora Colledge, Sophie De Massougnes, Bernice Elger

**Affiliations:** 1 Department of Sport, Exercise and Health, Birsstrasse 320B, 4052 Basel, Switzerland; 2 Hôpital ophtalmique Jules-Gonin, Avenue de France 15, Case postale 133, 1000 Lausanne, Switzerland; 3 Institute for Biomedical Ethics, Bernoullistrasse 28, 4055 Basel, Switzerland

**Keywords:** Ethics committee, Institutional review board, Informed consent

## Abstract

**Background:**

In Switzerland, research with identifiable human tissue samples, and/or its accompanying data, must be approved by a research ethics committee (REC) before it can be allowed to take place. However, as the demand for such tissue has rapidly increased in recent years, and biobanks have been created to meet these needs, committees have had to deal with a growing number of such demands. Detailed instructions for evaluating every kind of tissue request are scarce. Committees charged with evaluating research protocols therefore sometimes face uncertainty in their decision-making.

**Methods:**

We examine how a pool of Swiss REC members deal with a number of cases involving human tissue, in order to determine the standards they adhere to, and their understanding and implementation of existing laws and guidelines.

**Results:**

There is considerable divergence in the approaches and decisions of Swiss REC members regarding human tissue sample requests, particularly concerning the issue of informed consent. Despite recent trends towards less strict consent requirements for biosample research, many of our respondents continue to employ demanding standards for researchers. The question of informed consent, and the circumstances in which it is required, continues to result in differences of opinion.

**Conclusions:**

While room for local and cultural interpretation is essential to the workings of an REC, misunderstanding of existing guidelines, or an absence of regulation in sensitive areas, will only lead to suboptimal functioning of the REC itself. Our data suggests that there is uncertainty and disagreement on the question of consent for human tissue sample, which existing laws and guidelines may not fully clarify. Methods to address these uncertainties should be implemented in order to ensure efficient and harmonious review of research protocols.

**Electronic supplementary material:**

The online version of this article (10.1186/s12910-018-0331-0) contains supplementary material, which is available to authorized users.

## Background

Research involving human tissue, with or without accompanying clinical data, is currently regulated in Switzerland by a number of sources. The most specific is the Law on Research on Human Subjects (Humanforschungsgesetz), which addresses tissue samples in Articles 32 to 35 [1]. The Swiss Academy of Medical Sciences (SAMS) has developed a full set of guidelines outlining best practices for biobanks, and these also offer some guidance on work with tissue samples [2]. Elsewhere, the Federal Law on Pharmaceuticals and Medical Products [3], and the various cantonal laws, also apply to some aspects of biosample research.

While the SAMS and existing European guidelines [2, 4, 5] and domestic law provide some important information for researchers, and the ethics committee members who must regulate this research, the rapidly evolving nature of the field, coupled with the increase in demand for tissue samples, means that there are still unclear issues arising [6]. How exactly should the degree of anonymisation of the samples affect the ethical review process [7]? What sort of consent form must be obtained for human tissue samples [8–13]? Should results be returned to donors, particularly if a potentially dangerous diagnosis is uncovered [14]? These matters are still widely debated in the literature, yet they are crucial for research ethics committees (RECs) in their assessment of research protocols.

Studies assessing the decision criteria of RECs are scarce [15] and do not specifically address research involving biological samples. Our study is an initial assessment of the approaches of Swiss REC members to research protocols involving informed consent to human tissue sample use. Informed consent refers to the consent form which all Swiss ethics committees require for prospective studies. This is, except in exceptional cases, provided in written form. In the case of retrospective studies, the SAMS requires consent for the use of samples which are not fully anonymized. These requirements are identical for all committees in Switzerland. 7 RECs are responsible for all 26 cantons, and meet on a fortnightly basis (typically) to make decisions on applications for research projects involving human subjects or material. Committees are made up of a number of experts, with backgrounds ranging from medicine, to law, to nursing, to psychology, although for each application, only a small number of experts from this pool will be involved.

The aim is to understand how the committees and their members respond to such requests, whether responses differ between committees and their members, and some of the reasons for their approval or rejection of various parts of the study protocol. By understanding the ways in which decisions are made, and what areas produce uncertainty, it is possible to uncover issues which may require more discussion and clarification for REC members and researchers. The aim of our study was not to obtain statistically representative results, but to explore broadly which issues are the most controversial and why. We therefore adopted a qualitative approach, asking participants to provide comments, in order to understand the reasoning behind the decisions of the committee members. The study is also a didactic means to encourage further discussion of the addressed issues.

## Methods

After discussions with a number of Swiss ethical and legal experts and researchers involved in biobanking, a questionnaire was created based upon three fictional case examples, with several subsequent questions. These examples were designed specifically for the study to address various aspects of the informed consent process that were of particular interest to the researchers. These case examples, and the questionnaires which follow them, have not been validated. In Switzerland, research which is anonymised and not related to health or medical issues does not require ethical review [1]; consequently, no ethical review process was undertaken for this study.

The first case was followed by six closed and four open questions, the second by eight closed and six open questions, and the third by nine closed and eight open questions (see Additional file 1 for all questions.) Responses were given using a 5-point Likert-type scale (certainly agree, probably agree etc. to certainly disagree) [16], and we asked participants to provide comments after each question explaining the reasons for their responses. Agreement was categorized as a response of either “certainly” or “absolutely”, while disagreement was defined as a response of “probably not” or “certainly not”. Responses of “I don’t know” were not categorised as positive or negative, but counted separately. The aim was to support quantitative findings with qualitative data on the reasons for respondents’ decisions. This was felt to be an essential part of this study, as clarity about the reasons for possible misunderstandings or divergent answers are necessary for the improvement of future laws and guidelines. The case studies were designed to prompt responses on the committees’ approach to proposed research using human data and tissue.

Committees were identified via online searches for RECs in Switzerland. The presidents of these committees were contacted via email, provided with information about the nature of the study, and invited to take part. Committee presidents who agreed to do so received an information sheet about the study, outlining the requirements and aims, which they distributed to all individual members. Questionnaires were sent to the presidents of all working RECs in the French speaking parts of Switzerland While the cantons of Valais and Vaud have one cantonal commission with or without subsections, Geneva was at the time of the study composed of 4 research ethics (sub)committees supervised by one central commission at the University Hospitals of Geneva, and we included all 4 subcommittees. At the time of the study, the joint commission of the cantons Neuchatel, Fribourg and Jura was under reconstruction and not available and was therefore not included. One phone call and one follow up email were used as reminders.

Three case study questions were used to introduce the issues to participants. The first concerns [1] a request for tissue sample without accompanying data. The second [2] concerns a request for tissue with accompanying data. The third [3] deals with the drafting of a biobank’s written consent form. Questionnaires were completed either on paper and returned by post, or scanned and returned via email. Participants were informed that they were under no obligation to complete the questionnaire, and that the data would be anonymized; completing the questionnaire was taken as consent to participate. Upon receipt of all completed questionnaires, the primary investigator entered all data into a coded spreadsheet, with a single hard copy of the code key maintained in the possession of another investigator. Completed questionnaires were analysed by all authors. Responses to closed questions were grouped according to Likert scale levels. For open questions, two researchers independently analysed all responses and grouped them into categories based on theme and the nature of response (in many cases, for example, whether the response was positive or negative). Both researchers then compared findings and, in case of disagreement, asked a third member of the study team to resolve the issue.

## Results

Overall, we received a total of 31 completed questionnaires: 22 members of committees from Geneva, 6 from Vaud, and 2 from Valais responded. One commission president opted to discuss the questionnaire during a committee meeting and to provide one single response for the entire REC, while in the other committees, participants answered the questionnaire individually and anonymously. Consequently, the collective answer of this committee are counted as one response, as only one questionnaire was returned. This means that differences of opinion among the members of this committee cannot be determined; the data from this group response are valuable in light of the comparison between committees within the French-speaking part of Switzerland. Intra-committee disagreement is reflected by other committees.

We include some totals representing the results of the Likert scale questions not to make representative quantitative conclusions, but in order to indicate which issues where particularly controversial among respondents. In what follows, we present these results in particular to discuss the corresponding commentaries that elucidate reasons explaining the controversies.

### Case 1: Must a request for tissue for research purposes without accompanying data be approved by the entire REC? (see Additional file 1: Case 1)

The majority of respondents (25 out of 31) replied that, according to the recommendations followed by their committee, such a request would need to be approved by the committee as a whole, not fast-tracked (i.e. decided by the president only).

With regards to the open questions, respondents mentioned that the president alone was not the appropriate judge of such requests, and that the study protocol still had to be carefully verified.

### Would you approve the project if the sample were irreversibly anonymised?

A total of 21 out of 31 participants responded in favor of this proposition, with only four strongly opposing it. Respondents were also asked whether the patient’s consent would be required in such a case. Twelve respondents stated that consent would be necessary, 16 that it would not be necessary, while the rest were undecided.

From the qualitative questions, of those who felt that consent was not necessary, the decisive factor was the lack of risk to the patient: “No risk of misuse”; “No benefit or risk to donor”; “Unnecessary and costly administrative procedure”. Those who said that consent ought to be obtained felt that “regardless of study type, the patient must be informed”, and that “a biopsy is the patient’s property”.

### Would you approve the project if the samples were reversibly anonymised (i.e. identifiable via a code)?

Elaborating on the previous question, participants were asked whether the degree of anonymisation was the decisive factor in their approval of a project. In this case, 22 out of 31 respondents wrote they would still approve the project, although notably, more chose the “probably” option than in the previous question.

### Case 2: Must a request for tissue for research purposes with accompanying data be approved by the entire REC?

There was virtual unanimity (29 out of 31 in favour) on the fact that such samples, regardless of the degree of anonymisation, must be approved for use by the entire committee.

### Is the consent of the donor necessary for such a tissue request?

A total of 18 respondents stated that consent was required if the tissue was irreversibly anonymised, and 11 felt that it was, at least probably, not necessary. For the use of identifiable samples, 27 said consent would be required, with only two stating that it might not be necessary.

When asked to explain their views on both cases in the open questions, most held that consent was an automatic requirement for sample use. One participant stated: “Quality and results depend on the cooperation of the donor, which is only possible if he has consented”, while another wrote “If the samples are not irreversibly anonymised, the patient must be asked if he wishes to receive results, and potentially, bad news.” However, another respondent felt that “There are situations in which it is preferable that the patient not be contacted.”

### Should the discovery of a potentially bad health outcome be shared with the donor?

A total of 23 respondents thought that at least probably, such a discovery should be shared with the donor. Four disagreed, and four were unsure.

Based on the qualitative data, it appears that many of those who felt that the information should be shared commented on the fact that this must be made clear, and established, at the time of obtaining consent. Those who felt that perhaps the patient should not be contacted cited the possible unreliability of the study, and indeed the patient’s desire. The respondent had apparently assumed that the patient was aware of this possibility, and therefore agreed, in principle, that results should generally be returned.

### Case 3: Do RECs find a multiple choice consent form acceptable?

A total of 12 out of 31 respondents reported that they were probably or certainly opposed to a multiple choice consent form (see Additional file 1: case 3, highlighted in the box), while 15 would at least probably accept it. An examination of the data reveals that in two separate cases, members of the same REC held diametrically opposing views.

### Should the choice permitting the samples to be used in any future medical research appear on the form (blanket consent)?

An almost even split was found for responses to this question, with 14 respondents opposing the suggestion, 16 in favour, and one unsure.

When explaining their responses, respondents who opposed this formulation of a **blanket** consent felt that the patient would not be sufficiently informed to make a proper decision. Interestingly, they indicated that a blanket consent was practically impossible, not simply undesirable. Among the comments on this proposal were: “Too vague”, “Insufficient information for the patient”, “Prior consent would be required”, and “The protocol must describe the specific goals of the research”. One respondent cited the SAMS guidelines on biobanking stating that Article 4.3 of the guidelines does not permit blanket consent (however the respondent apparently had a different understanding of the guidelines, as in fact, the article does allow for a “general” form of consent encompassing unspecified future uses).

Of those in favor of blanket consent, only one respondent gave a reason: “Patients in a hospital must expect their samples to be used in future research unless they sign a form refusing this.” On this point, in three separate cases members of the same committee gave diverging responses.

### Should the choice permitting the samples to be used for research on colorectal cancer appear on the form?

Four respondents opposed this suggestion, will two were undecided.

Based on the qualitative data, respondents in favor of this proposition approved of the fact that it limited the scope of the research to a specific domain, and that it was explicit enough to ensure that the donor would know what he was agreeing to. One respondent explained “It seems sensible to me that if one goes to the trouble of creating a biobank, one ought to be able to use the samples for any aspect of colorectal cancer research, with the proper REC approval.”

Those who opposed this choice stated, once more, that it was too broad. One respondent felt that it would be sufficient to indicate that the intended research was in the field of genetics. Others felt that this option was still too vague to enable the donor to be properly informed in his consent, though this option seemed to be generally more acceptable than the previous one..

### Should the choice permitting the samples to be used for research on the APC gene appear on the form?

Again, four respondents opposed this suggestion, with two undecided, leaving twenty-five in favour. As justification for these choices, certain respondents reported that this choice was “vague”, with one stating that “One must always specify the exact details of the research project,” and another stating that even research on a specific gene might have many aspects which the donor would not be aware of.

Others felt that this provision was unduly limiting, as “it would require the researcher to re-contact his patients if he wished to perform further research” and “it’s restrictive: one could discover useful things on colon cancer.” One committee showed that two members held opposing views on this topic.

## Discussion

Discussions about criteria for ethics approval of research involving tissue samples have been a feature of literature in a theoretical form for some time. The uniqueness of our study is that we have used detailed case examples and solicited the opinions of REC members about whether and under which conditions the projects should be approved. This debate can only be meaningfully advanced if the discussion takes into account real-world situations, and includes the actors directly involved in the decision making.

For these reasons, a particularly important finding of our study is that there is considerable divergence in the approaches and decisions of Swiss REC members regarding human tissue sample requests. In some cases, only a few members of certain committees express different viewpoints, or raise questions concerning the examples used. However, on a number of issues, there are significant differences in approach. Some differences may be due to cantonal or institutional regulations; while all committees in Switzerland must ensure that the same national and international laws are upheld, many research applications come from universities. These frequently differ with regards to data storage and sharing policies, and may face logistical challenges, such as lack of sample storage space or cooperation with international partners, which present challenging situations for the relevant RECs. More importantly, our results also demonstrate that members of the same cantonal committee hold opposite views. Divergence might be caused by a lack of knowledge, understanding, or clarity of laws and guidelines and would point to a need for more specific training and clarification from the inter-cantonal REC association (AGEK, Swissethics). These being the areas which can and must be improved on in order to allow optimal REC functioning, it is essential to understand their extent. Inconsistency and disagreement in RECs, both internal and between independent committees, can slow research, and can result in varying interpretations of laws and regulations which directly affect research participants [17, 18]. Our findings are an initial indication of areas which require some harmonization efforts.

The most notable finding of our study is that all three case examples, the greatest areas of disagreement concerned informed consent. Below, we discuss the points which produced significant differences, and suggest ways in which this could be minimized.

Questions related to case 1 revealed that a high level of disagreement existed between committee members on whether research involving irreversibly anonymised samples, with no accompanying clinical data, would require the informed consent of the donor. The SAMS guidelines [2] state, in section 4.3, that no express consent is required for such research. Irreversibly anonymised samples are widely held to pose virtually no risk to donors when used in research, and it is frequently argued that this lessens the need to obtain fully informed consent [19, 20]. Hence, requests for consent by RECs risk slowing the progress of research [12, 21]. It may be that respondents who stated that they would refuse such requests are not frequently confronted by requests pertaining to tissue samples, and apply the criteria that governs research on humans subjects.

While at the time of the survey the Law on Human Subject Research was not in effect, it is worth noting that its current wording may still be perceived as ambiguous by those tasked with upholding it. Informed consent is not required if obtaining it would be impossible or very difficult, or if the ends of the research outweigh the interests of the donor; it may be the case that specific, case-based examples would facilitate the decision-making process for REC members who are tasked with finding the appropriate balance between research and patients interests. Furthermore, the SAMS guidelines emphasise that inappropriate irreversible anonymisation should be avoided: “Both in the interests of the patients and in the interests of research, samples and data should not be irreversibly anonymised, as far as this is possible. For the patient, irreversible anonymisation means that generally he can no longer be informed of relevant results; for research, it means that the samples and the data lose in informative value” [2](p.6).

Regarding case 2, the most noteworthy result is that almost two-thirds of respondents are convinced that consent is necessary for samples accompanied by clinical data, regardless of whether or not they were irreversibly anonymised. When comparing case 1 to case 2 it seems that the existence of accompanying clinical data is a motivating factor in the need for obtaining consent. Whether committee members feel that this increases the risk (which will depend on the richness of the data and the procedure of anonymisation), or that it increases the chance that useful results are found (a realistic possibility, given the views on return of results) is not clear. As noted above, the SAMS guidelines, and the Declaration of Helsinki [22], do not make such a requirement. The Law on Human Research now makes clear that this is not always the case, which may contribute to resolving disagreement on this point.

Case 3 gives some of the most detailed explanation of respondents’ decision making, and provides insight into Swiss committee members’ approaches to the divisive field of consent in biobanking. Informed consent procedures typically receive a great deal of focus in ethics review [23]. In Switzerland, informed consent is protected, though not explicitly, by Article 10 of the Swiss constitution [24] and Article 28 of the Civil Code [25]. As well as being a crucial aspect of every international declaration on health care ethics since the Nuremberg Code, it is defended by Article 1 of the SAMS manual on research on human subjects [26]. Switzerland therefore operates under a legal and ethical framework which recognizes the primacy of informed consent in medical research.

Of the individual consent options (which could, but must not necessarily, form part of a multiple choice form), a majority of respondents opposed the notion of a blanket consent. An even greater majority, however, were in favor of the two more specific options for consent. The more restrictive option (APC gene research) was no more favored than the slightly broader one (colorectal cancer research). Interestingly, those respondents who would accept a multiple choice form would in most cases also accept a more broad consent. These options represent a somewhat enlarged approach to consent in comparison to the traditional human subject requirements, so the correlative acceptance of both ideas is perhaps to be expected.

Our results show that only about half of the respondents approve of these more open approaches to consent, demonstrating that this remains controversial among the participating REC members. According to the Law on Human Subject Research, biological material may be reused in coded or non-coded form if the sample source has consented after having received sufficient information. The exact definition of how general or detailed the information may be in order to be considered sufficient will remain at the discretion of individual RECs. This is likely to be a key factor in the disagreements reported, as it represents an issue free of strict regulation, in which the experiences and perhaps the local context of committee members (such as the research focus on the universities they represent) take on a prominent role in decision making. It may, however, still be desirable for RECs to work towards harmonization on this matter, as any study which requires a multi-centric project to be approved by various committees risks confusion and potentially a stalled or blocked application.

Our results indicate that at present REC members prefer to err on the side of restriction as many respondents have chosen to adhere to the traditional form of consent used in clinical trials. However, there seems to be growing agreement that the specifics of the study protocol can affect the level of consent and the details of information deemed necessary. Clearly, the interpretation of the SAMS guidelines and the Law on Human Research should be clarified in further training for REC members. Indeed, the guidelines do not prohibit blanket consent, as one respondent claimed, but might require more specific consent, if the primary investigator is aware of his planned research and reuse of the samples before he takes the samples. Here again, if the sample is part of a tissue extraction undertaken for treatment purposes, the need for specific consent may not arise (for a full discussion of the intricacies and different scenarios which can occur, see Junod and Elger, (2010) [1]).

Finally, it should be noted that in case 3, on a number of occasions, members of a single ethics committee give different responses as to how their committee operates. This represents a noteworthy lack of agreement about the very policies and practices of the committee. If not all members of one committee agree about how protocol review should be handled, transparent criteria need to be used to resolve the inconsistencies. Our study highlights the need for committees to work closely, and together with committees from other cantons, in order to ensure a well-argued and transparent level of ethical review.

Disagreement between REC members and different RECs [15] occurs for three main reasons. Different committees may assess risks and benefits using particular paradigms, or may assign different worth to certain values, for example patient autonomy versus public good [23]. This is an integral part of the ethical review process itself, allowing for consideration of local norms and standards, and detailed analysis of the need for research in that particular time and place. However, as addressed above, disagreement can also be caused by a lack of knowledge or a variable interpretation of relevant laws and guidelines (or indeed, the details of the protocol), or by an absence of such documents. In all of these cases, decisions appear to be influenced factors which have no solid basis in current law, hence differences of this kind should be minimized as far as possible. However, it is important to bear in mind that guidelines and laws as well as their interpretation may change over time, and are also sometimes left deliberately vague. Legislators may have purposely left certain parts of the law open to interpretation to allow for a degree of variation and adaptation to local culture or circumstances. The lack of specificity may, however, have the secondary effect of leaving committee members with insufficient guidance [15].

Our results indicate that there are some differences in the decisions made by Swiss ethics committees which cannot be explained simply by regional differences. Particularly important are the approaches to requiring informed consent for unidentifiable samples, and the various views on the level of consent necessary when tissue samples are obtained. We have suggested that this may be a result of the fact that traditional paradigms of informed consent are giving way to more relaxed recommendations in the case of tissue samples, and that certain ethics committees have yet to “catch up” with these changes.

It is essential that ethics committees in Switzerland are fully aware of the laws which will affect their assessment of study protocols, and that all members of the committee are kept up to date with new developments. A Switzerland-wide training and information programme for committee members would be one way of working towards this [27]. Consultation with committees on the optimum way to transmit such information would also be a useful step. It has also been suggested that ethics committees could create sub-groups with specialisms in certain types of study [15].

### Limitations

Our study has several limitations. We have included only RECs from one language region in Switzerland; hence, this study is not representative of all of Switzerland, but merely serves an example of some of the issues encountered one part of country. Due to the higher number of REC members in the subcommittees in Geneva, the opinions of REC members in this canton are overrepresented. On the other hand, this represents to some extent the charge of protocols: RECs in cantons with university hospitals treat a much higher number of protocols than RECs in cantons without a university hospital. Furthermore, our aim has not been to present a quantitative overview about opinions in Switzerland but to explore agreement and disagreement among REC members of the same committees and across some other cantons. The research design, including the qualitative analysis of the comments, has enabled us to show significant controversy. However, the present data does not allow for any understanding of the reasons for disagreement or difference in interpretation between committee and their members, and we recommend this as a topic for future studies. Another limitation is the hypothetical nature of the cases. We did not evaluate how RECs would react to real protocols and answers could be influenced by social desirability. However, we ensured strict anonymity of the answers, and given their sometimes controversial tone, we have no indication for a one-sided social desirability bias.

## Conclusion

As samples of human biological material become increasingly widely used in research, the traditionally strict requirements for informed consent are becoming more flexible, reflecting the potential benefits of such research, and the minimization of physical risk to human donors. Switzerland’s Law on Human Subject Research and changes to the Helsinki Declaration [28] reflects this trend. At present, there is a division in Swiss ethics committees on a number of issues concerning the use of human biosamples. While this division perhaps is to be expected at a time when regulations are changing, differing standards within RECs and between cantons are undesirable. They may be confusing to researchers, hamper prospective studies, and even contravene accepted guidelines. In addition, certain cantons may develop a reputation as being “easy to please”, while others will be avoided for their strict regulations, with potentially disruptive effects in scientific research in Switzerland. Our results show that discrepancies do exist across all participants, i.e. between cantons as well as within committees. Education and further training of researchers and committee members on ethical and legal issues surrounding research involving biobanks would be helpful to clarify uncertainties and to support timely and harmonized research review. For example, a half-day course summarizing relevant legal requirements and standards put in place by international guidelines, similar to good clinical practice training, could contribute towards more clearly informed decision-making. A monthly handout, produced by the SAMS and summarizing new developments or frequently asked questions, could also be a valuable and not excessively demanding way of keeping committee members informed.

## Additional file

Additional file 1: Case study boxes 1, 2, 3. Case studies and questions given to participants. (DOCX 16 kb)
