# Correction to: Consent requirements for research with human tissue: Swiss ethics committee members disagree

**DOI:** 10.1186/s12910-019-0365-y

**Published:** 2019-04-18

**Authors:** Flora Colledge, Sophie De Massougnes, Bernice Elger

**Affiliations:** 1 Department of Sport, Exercise and Health, Birsstrasse 320B, 4052 Basel, Switzerland; 2 Hôpital ophtalmique Jules-Gonin, Avenue de France 15, Case postale 133, 1000 Lausanne, Switzerland; 3 Institute for Biomedical Ethics, Bernoullistrasse 28, 4055 Basel, Switzerland

**Correction to: BMC Med Ethics (2018) 19:93**

**https://doi.org/10.1186/s12910-018-0331-0**

It has come to our attention that in the original article [1] information regarding dates was omitted. The data in this study were obtained in Switzerland four years before the entering into force of the new Swiss Human Research Act in 2014, when the guidelines of the Swiss Academy of Medical Sciences (SAMS) ceased to apply. It is important for readers to know that at the time of the study there was no binding law in Switzerland, only the more open SAMS guidelines that have a different legal status. We would expect to find less variation of opinions among research ethics committee members if the study were repeated after the federal law came into force.
