# Supplementary material for: Consent requirements for research with human tissue: Swiss ethics committee members disagree
Source: BMC Med Ethics. 2018 Nov 26;19:93. doi: 10.1186/s12910-018-0331-0 (PMC6260886; doi:10.1186/s12910-018-0331-0)
Supplement: Supplementary file 1 — Case study boxes 1, 2, 3. Case studies and questions given to participants. (DOCX 16 kb) [file 12910_2018_331_MOESM1_ESM.docx]

*Box 1: request for tissue sample without accompanying data.*

| Case 1 |
| --- |
| A researcher working in the gynecological department of a university hospital is undertaking a study on the efficacy of chemotherapy on breast cancer. He/she has noticed that in some cases chemotherapy is very successful, while in other cases it seems to have no effect on the tumor, and maybe be nothing but dangerous for the patient due to its side effects.  He/she wishes to carry out a comparative study between cases responding well to treatment and cases showing no response, in order to potentially identify markers for tumor response to chemotherapy.  He/she contacts the head of the pathology institute explains the project, and asks for 50 samples of anonymised mammary carcinoma, 25 of which responded successfully to treatment, 25 of which did not. To ensure that the samples are comparable, he/she asks the pathologist to provide tissue At the same TNM stage. For this study, the researcher does not require the patients’ medical records or information. |

1a Based on the rules and laws currently in force, must the study in question go through the normal channels for a fully study approval (i.e. no expedited process, all committee members involved)?

Certainly___ Probably___ I don’t know___ Probably not___ Certainly not___

1b. In your personal opinion, would it be possible for this study to go through an expedited approval process?

Certainly___ Probably___ I don’t know___ Probably not___ Certainly not___

Please explain your response

1c. If this project does not need to undergo a full ethical approval, what process is applied in your committee?

Please explain your response

1d. Would you approve this study if the samples were irreversibly anonymized, that is, no link to patient data was maintained?

Certainly___ Probably___ I don’t know___ Probably not___ Certainly not___

1e. in the case of irreversibly anonymized samples, do you think that patient consent is still required?

Certainly___ Probably___ I don’t know___ Probably not___ Certainly not___

Please explain your response

1f. Would you approve the study if the samples were reversibly anonymized, that is, the pathologist would still be able to identify each donor patient?

Certainly___ Probably___ I don’t know___ Probably not___ Certainly not___

1g. If so, is the consent of the patient necessary?

Certainly___ Probably___ I don’t know___ Probably not___ Certainly not___

Please explain your response

*Box 2: request for tissue with accompanying data.*

| Case 2 |
| --- |
| A colleague of the previous researcher is also interested in factors related to breast cancer. He/she hypothesizes that certain tumours discovered during mammograms are stable, and, if left untreated, they would only rarely evolve into invasive carcinomas. In line with current treatment protocols, all suspected tumors are biopsied, and he/she would like to carry out a study on the possible difference between tumors in terms of malignancy.  He/she contacts the pathologist, tells him about the project, and asks for 100 samples of breast tumor, diagnosed in the last 5 years. He/she also asks for access to the medical records in order to obtain information about the samples, such as the size of the tumor five years ago, the development of the case (recurrence, new tumor, death), the patient’s age, if signs for other cancers are present, and family history.  The researcher also thinks that if he/she discovers predictors for malignancy, indicating, for example, risks of recurrence, it would be best to inform the patient. With this in mind, he/she asks the pathologist for reversible anonymised samples only, in order that the patient could be identified by the pathologist if necessary. |

2. For the following questions, please respond based on this information: Based on the rules and laws currently in force, must the study in question go through the normal channels for a full study approval (i.e. no expedited process, all committee members involved)?

2a. With irreversibly anonymized samples.

Certainly___ Probably___ I don’t know___ Probably not___ Certainly not___

2b. With reversibly anonymized samples

Certainly___ Probably___ I don’t know___ Probably not___ Certainly not___

2c. If this study does not require full ethical approval, what process is applied in your committee?

For the following questions, please respond based on this initial question: Does this study require the patient’s consent?

2d. With irreversibly anonymized samples.

Certainly___ Probably___ I don’t know___ Probably not___ Certainly not___

2e. with reversibly anonymized samples

Certainly___ Probably___ I don’t know___ Probably not___ Certainly not___

Please comment on questions 2a-e

2f. The primary investigator in this study discovers a predictive factor for rapidly developing invasive tumours. Do you think that patients whose samples were used in this study should receive this information if this factor is present in their samples, although no developed carcinoma in situ has been observed?

Certainly___ Probably___ I don’t know___ Probably not___ Certainly not___

Please explain your response

2g. What process in your committee governs the communication of such information?

2h. Would you approve this study if the samples were irreversibly anonymized.

Certainly___ Probably___ I don’t know___ Probably not___ Certainly not___

2i. Would you approve the study if the samples were reversibly anonymized.

Certainly___ Probably___ I don’t know___ Probably not___ Certainly not___

2j. The primary investigator in this study discovers a predictive factor for rapidly developing invasive tumours. However, this finding has not been confirmed by a subsequent study. Do you think that patients whose samples were used in this study should receive this information if this factor is present in their samples, although no developed carcinoma in suit has been observed?

Certainly___ Probably___ I don’t know___ Probably not___ Certainly not___

Please explain your response

2k. In your committee, who is a decision reached concerning the information/findings which should be communicated to study participants?

*Box 3: drafting of a biobank’s consent form*

| Case 3 |
| --- |
| A surgeon, who is also the head of a laboratory, intends to undertake a project on colorectal cancer, and its development, including initial stages, genetic risk factors, metastatisation, etc.  For the project, he/she would like to develop a broad consent form for all patient hospitalized for surgery, in order to build a biobank in collaboration with the pathology institute. He seeks permission from the local ethics committee.  Currently, the surgeon wants to use samples and clinical data for a project on the APC gene, linked to colorectal cancer, and in future, he7she would like to use these samples for unspecified projects. In order to minimize the burden on patients, he/she would like to present them with the following consent form:  “ I consent to my sample(s) and clinical and personal data being stored, so that they may be used for (please tick all that apply):  __Any medical research.  __Medical research into colorectal cancer.  __Research on the APC gene.” |

3a. Would you accept the surgeon’s demand to create biobank?

Certainly___ Probably___ I don’t know___ Probably not___ Certainly not___

3b. Do you think a consent form using multiple choice is acceptable?

Certainly___ Probably___ I don’t know___ Probably not___ Certainly not___

If not, which of the three options listed below should be a part of the consent form:

A The patient accepts that his/her samples will be stored and used for future medical research projects of any kind.

Certainly___ Probably___ I don’t know___ Probably not___ Certainly not___

Please explain your response

B The patient accepts that his/her samples will be stored and used for research on colorectal carcinoma only.

Certainly___ Probably___ I don’t know___ Probably not___ Certainly not___

Please explain your response

C The patient accepts that his/her samples will be stored and used for research on the APC gene only.

Certainly___ Probably___ I don’t know___ Probably not___ Certainly not___

Please explain your response

3c. The surgeon prefers not to inform the patient about the results, and states clearly at the end of the consent form: “ patients will not be informed about the results of studies in which their samples are used.” Is this acceptable?

Certainly___ Probably___ I don’t know___ Probably not___ Certainly not___

Please explain your response

3d. A member of your committee suggests a new addendum to the consent form: “ If, in the course of research projects involving your samples, we discover information which could be relevant to your health, would you like to be informed?” Is this acceptable?

Certainly___ Probably___ I don’t know___ Probably not___ Certainly not___

Please explain your response

3e. Another committee member proposes a different addendum: “ If, in the course of research projects involving your samples, we discover information which could be relevant to your health, this will be communicated to you, either directly or by your treating physician. The relevance of the information will be evaluated by an ethical committee.” In your opinion, is this acceptable?

Certainly___ Probably___ I don’t know___ Probably not___ Certainly not___

Please explain your response

3f. the protocol specifies that part of the genetic screening will take place in the United States. The researcher does not this it is necessary to inform patients that their samples will be shipped overseas. Is this acceptable?

Certainly___ Probably___ I don’t know___ Probably not___ Certainly not___

Please explain your response
